# Evidence of premature vascular dysfunction in young adults who regularly use e-cigarettes and the impact of usage length

**DOI:** 10.1007/s10456-023-09903-7

**Published:** 2024-02-12

**Authors:** Chloe Matheson, Tijana Simovic, Allison Heefner, Marisa Colon, Enrique Tunon, Kolton Cobb, Christopher Thode, Alison Breland, Caroline O. Cobb, Patrick Nana-Sinkam, Ryan Garten, Paula Rodriguez-Miguelez

**Affiliations:** 1 Department of Kinesiology and Health Sciences, Virginia Commonwealth University, 817 West Franklin Street, Richmond, VA 23284 USA; 2 School of Medicine, Virginia Commonwealth University, Richmond, VA USA; 3 Department of Psychology, Virginia Commonwealth University, Richmond, VA USA; 4 Division of Pulmonary and Critical Care, Virginia Commonwealth University, Richmond, VA USA

**Keywords:** E-cigarettes, Tobacco products, Microvasculature, Vascular health

## Abstract

**Background:**

Electronic (e-) cigarettes are increasingly popular tobacco products on the US market. Traditional tobacco products are known to cause vascular dysfunction, one of the earliest indicators of cardiovascular disease (CVD) development. However, little is known about the effect of regular e-cigarette use on vascular function. The purpose of this study was to investigate the impact of regular e-cigarette use on vascular function and cardiovascular health in young, healthy adults.

**Methods:**

Twenty-one regular users of e-cigarettes (ECU) and twenty-one demographically matched non-users (NU) completed this study. Vascular health was assessed in the cutaneous microcirculation through different reactivity tests to evaluate overall functionality, endothelium-dependent vasodilation (EDD), and endothelium-independent vasodilation (EID). Macrovascular function was assessed using flow-mediated dilation (FMD).

**Results:**

Our results suggest that regular users of e-cigarettes present with premature microvascular impairment when compared to non-users. Specifically, they exhibit lower hyperemic (*p* = 0.003), thermal (*p* = 0.010), and EDD (*p* = 0.004) responses. No differences in EID between the groups were identified. We also identified that individuals who use e-cigarettes for longer than 3 years also present with systemic manifestations, as observed by significantly reduced macrovascular (*p* = 0.002) and microvascular (*p* ≤ 0.044) function.

**Conclusions:**

Our novel data suggests that young, apparently healthy, regular users of e-cigarettes present with premature vascular dysfunction in the microcirculation when compared to non-users. We have also identified systemic vascular dysfunction affecting both the micro and macrovasculature in those young individuals who used e-cigarettes for longer than 3 years. Taken together, these findings associate regular e-cigarette use with premature vascular dysfunctions and adverse cardiovascular outcomes.

## Introduction

Electronic nicotine device systems, commonly known as electronic (e-) cigarettes, or vapes, are battery-powered devices that deliver vapor containing a flavoring component and nicotine. These products officially entered the U.S. marketplace in 2007 and since then, their use has rapidly increased due to their popularity among the younger generations [1]. While e-cigarettes are often marketed as a safer alternative to traditional cigarettes [2], their impact on cardiovascular health is still a topic of active research.

The association between traditional tobacco use and increased cardiovascular risk is well-documented, with tobacco being recognized as one of the major contributors to heart disease. Early observations, primarily from preclinical models, have linked e-cigarette usage with different cardiovascular disease risk markers [3–5]. More recent studies have also shown that acute exposure to e-cigarettes may impact cardiovascular health in humans [6, 7] causing reduced vascular function [8–10] and an associated increase in oxidative damage [8, 11]. Comparable results have also been identified in the macrovasculature of chronic adult users of e-cigarettes, with a similar dysfunction as that observed in traditional tobacco users [12], although opposite results have been also described [13–15].

Microvascular function plays a pivotal role in cardiovascular health and dysfunctions in the microcirculation are considered among the earliest indicators of cardiovascular disease risk that can be identified prior to traditional clinical markers [16]. In fact, microvascular dysfunction is a crucial mechanism in the development and progression of cardiovascular disease and often precedes other macrovascular pathological processes [17, 18]. A recent study has shown reduced microvascular function, particularly endothelium-dependent, in chronic users of e-cigarettes [19]. Despite these early findings, the regular effects of e-cigarette usage on vascular function are still not yet well understood. Thus, this study aims to investigate the relationship between micro and macrovascular function, cardiovascular health, and e-cigarette usage in young adults. We hypothesized that regular users of e-cigarettes, who are otherwise healthy young adults, will exhibit reduced micro- and macrovascular function when compared to demographically matched non-users.

## Material and methods

### Experimental design

Volunteers presented to the Vascular and Integrative Physiology (VIP) Laboratory on two separate occasions: a preliminary day and an experimental day. The preliminary day consisted of assessing body composition, blood pressure, medical history, and overall health status. For the experimental day, participants reported to the VIP Laboratory following an overnight fast, having abstained from e-cigarette usage and caffeine for 12 h, vigorous physical activity for 24 h, and vitamin supplementation for 72 h. Vascular health through evaluation of macro- and microvascular function was completed.

### Participants

A total of forty-two young adult men and women, ages 21–31 years were enrolled in the present study, following the principles of the Declaration of Helsinki and after approval by the Institutional Review Board at Virginia Commonwealth University. Among the participants, 21 young adults were regular users of e-cigarettes (≥ 3 times/week for ≥ 3 months) while 21 young adults were non-users demographically matched considering age, sex, and body mass index. Participants from both groups were excluded if they used (1) cigarettes or other tobacco products (cigars, hookahs, smokeless) in the past 60 days, (2) any illicit or prescription drugs for non-medical used weekly or more frequently in the past 60 days, (3) were diagnosed with any cardiovascular, pulmonary, renal, hepatic, metabolic, and cerebral diseases, or (4) were currently pregnant or nursing.

Participants completed self-reported questionnaires, including the Short Form Vaping Consequences Questionnaire, E-Cigarette Dependent Scale, and the NIDA Quick Screen to report type of e-cigarette products or usage of other tobacco products as well as alcohol and drug use. Out of 21 regular users of e-cigarettes, 20 self-identified as sole users of e-cigarettes, never smokers, primarily using fourth generation e-cigarettes. All participants were informed of the objectives, and possible risks of the investigation before written consent for participation was obtained.

### Demographic characteristics

Demographic characteristics were evaluated in all the participants during the preliminary visit. Volunteers completed a standard anthropometric assessment of height, weight, and calculated body mass index (BMI). A self-reported evaluation of e-cigarette usage was obtained from each participant using the E-Cigarette Dependence Scale [20] and the Penn State Electronic Cigarette Dependence Index [21].

### Clinical laboratory values

A venous blood sample was collected for the assessment of a complete blood count and a comprehensive metabolic panel by standard core laboratory techniques (Laboratory Corporation of America Holdings, Burlington, NC). Fasting concentrations of lipids (total cholesterol (TC), high-density lipoproteins (HDL), low-density lipoproteins (LDL), and triglycerides) and glucose were obtained using the Cholestech LDX analyzer (Alere, Providence, RI). Hemoglobin and hematocrit values were obtained using the HemoPoint H2 analyzer (Stanbio Laboratory, Boerne, TX).

### Microvascular function

Microvascular function was evaluated using a laser speckle contrast imager (MoorFLPI2, Moor Instruments, DE) of the cutaneous circulation of the forearm. Briefly, the right arm of each participant was extended laterally, and the distal forearm was secured in a vacuum-packed pillow (Vacpac, MD). A forearm cuff was placed immediately distal to the medial epicondyle, and three chambers were placed on the ventral surface of the forearm. The placement was carefully selected, avoiding any area with hair, broken skin, areas of skin pigmentation (or tattoos), and visible veins.

After a 20 min acclimation period and in a temperature-controlled room (22 ± 2 °C) to achieve a hemodynamic steady state, baseline (B_L_) flux was determined by calculating a 30-s average. A biological zero (B_0_), to control for the Brownian movement of macromolecules in cutaneous interstitial space, was also determined during a reduction of blood flow in the forearm and subtracted from both baseline and peak responses. Then, four different reactivity tests and control were completed:

1. *Local thermal hyperemia* (LTH) was completed to determine maximal microvascular dilation [22] in all the participants. A chamber (Moor VHP3) was attached to the volar surface of the forearm and filled with 2 ml of deionized water and heated at > 0.1 °C/s to 44 °C for 25 min [22]. This protocol elicits a biphasic response with an initial dilation primarily mediated by axon reflex [23] and a second dilation primarily mediated by nitric oxide [23] and endothelial-derived hyperpolarization factors (EDHF) [24]. Maximal dilation achieved during this protocol was used to compare the relative response to other protocols [23]. All variables related to this protocol are defined with the subscript LTH.
2. *Post-occlusive reactive hyperemia* (PORH) was completed in all the participants to evaluate microvascular shear-stress response primarily mediated by sensory nerves and EDHF [25, 26]. A forearm cuff was inflated to 250 mmHg for 5 min. After the occlusion period, the release of the pressure elicited a hyperemic response. All variables related to this protocol are defined with the subscript PORH.
3. *Iontophoresis of acetylcholine* (ACh) to assess microvascular endothelial-dependent vasodilation through nitric oxide, EDHF, and prostaglandins mediators [22, 27]. A chamber with an internal platinum wire electrode (Moor MIC-ION 6) was attached to the skin of the volar aspect of the forearm by a double-sided adhesive disk filled with a solution of 2% ACh in 0.5% NaCl. The drug was delivered in the dermis using an incremental current delivery with three scans of 20 s at 5 µA, 10 µA, 15 µA, 20 µA, and 25 µA, giving a total charge of 4.5 mC. Short scan times and low currents were used to minimize non-specific vasodilation [22, 28]. All variables related to this protocol are defined with the subscript ACH. Due to technical issues, all participants minus two from the ECU group completed this test.
4. *Iontophoresis of sodium nitroprusside* (SNP) to evaluate microvascular endothelial-independent response through the delivery of nitric oxide to the smooth muscle cells [29]. A second chamber with an internal platinum wire electrode (Moor MIC-ION 6) was also attached to the volar aspect of the forearm. A solution of 2% SNP in 0.5% NaCl was used and delivered to the dermis using the same protocol previously described for Ach. All variables related to this protocol are defined with the subscript SNP. Due to technical issues with the delivery chamber, sixteen participants from the ECU and eighteen from the NU group completed this test.
5. *A non-reactive site* was monitored as a control site all the participants. An arbitrary area of the skin between the three chambers was used to evaluate non-specific vasodilation and/or movement for 30 min. All variables related to this assessment are defined with subscript C.

For each test, cutaneous blood flow was indexed as red blood cell flux (RBF) in perfusion units (PU) and as cutaneous vascular conductance (CVC) when RBF was controlled by mean arterial blood pressure. Results are presented as (1) the overall hyperemic response (Response), (2) the area under the curve (Area), (3) the relative change in flux expressed as a percentage of maximal dilation (%_max_) and (4) the time-to-peak (TTP) which represents the time from the start of the stimuli to the maximal hyperemic response. Skin resistance values were calculated from the recorded voltages using Ohm’s Law at each of the iontophoresis scans [30].

### Macrovascular function

Brachial artery macrovascular function was evaluated in all the participants using the flow-mediated dilation (FMD) test. A detailed explanation of the technique has been described previously [31]. Briefly, using a 12-MHz linear transducer, simultaneous B-mode and blood velocity profiles of the brachial artery were evaluated through ultrasound imaging (Logiq 9 XD Clear, G.E. Medical Systems, Milwaukee, WI). After an initial baseline, a forearm occlusion cuff (D.E. Hokanson, Bellevue, WA) placed immediately distal to the medial epicondyle, was rapidly inflated to 250 mmHg for 5 min (E-20 rapid cuff inflator, D.E. Hokanson,) to induce arterial occlusion. Then, the pressure of the cuff was released inducing reactive hyperemia of the brachial artery [31]. Automated offline analysis of brachial artery vasodilation was completed (FMD Studio, Quippu, Italy). Peak diameter was determined by the highest five-second average following cuff release. FMD is expressed as the percent increase in peak diameter from baseline diameter. The cumulative shear rate (area under the curve, AUC) was determined every 4 s for the first 20 s, and every 5 s thereafter for the remainder 2-min data collection period using the trapezoidal rule. FMD was normalized by shear rate and presented as FMD/shear [32]. Low flow-mediated constriction (L-FMC) was calculated to evaluate resting arterial tone as the percent decrease in diameter in the last 30 s prior cuff release and compared with baseline diameter [33]. A composite endpoint (c-FMD) was also calculated as the sum of the absolute values of FMD and L-FMC [33].

### Statistical analysis

The data were analyzed using SPSS version 29 (SPSS Inc., Chicago, IL) and expressed as mean ± standard error of mean (SEM) unless otherwise noted. An initial power calculation was performed based on the anticipated effect size estimated for the primary outcome (*n* = 15, power > 0.80). The initial proposed sample size yielded a power > 0.88 in the primary outcome for the present study (microvascular function). A power analysis and sample size calculation were performed before initiating the study, considering that under most circumstances, an *α* = 0.05 and a statistical power ≥ 0.80 is well accepted.

For all statistical analyses, significance was set at *p* < 0.05. The Shapiro–Wilk test was used to analyze the normality of the measurement distribution. When normality was met, independent group *t* tests were performed to identify group differences between users of e-cigarettes and non-users. If normality was not met, Mann–Whitney *U* tests were completed. Results are illustrated with box-and-whisker plots with minimum and maximum values. Effect size calculations using Cohen’s *d* were reported for primary outcomes to represent small Cohen’s *d* = 0.2), medium Cohen’s *d* = 0.5), and large Cohen’s *d* = 0.8) effect sizes [34, 35]. Relationships between vascular function and e-cigarette usage including frequency, length of usage, and nicotine content were evaluated using Pearson’s correlation coefficients (*r*). To further compare the effects of e-cigarette usage on vascular health, we divided e-cigarette users based on the median e-cigarette use duration (3 years) into those that have used e-cigarettes for longer than 3 years (> 3 year: *n* = 10) and those that have used these products for shorter than 3 years (≤ 3 year: *n* = 11).

## Results

### Participant characteristics

Demographic characteristics and clinical laboratory values for users of e-cigarettes and non-users are presented in Table 1. No differences in subject demographics and anthropometrics were observed between participants from both groups. Similarly, no differences were identified in the clinical laboratory values between the groups. As expected, significant (*p* ≤ 0.009) differences between the groups were identified in both nicotine and cotinine concentrations. Participants biological sex was considered during data analysis and no statistical differences were present between male and female participants.

**Table 1:** Participant characteristics and laboratory values of young users of e-cigarette and non-users

Variable	Users of e-cigarettes	Non-users	*p* value
*N*	21	21	–
Sex (M/F)	10/11	9/12	0.624
Age (years)	23 ± 3	25 ± 5	0.126
Height (cm)	172 ± 9	169 ± 9	0.313
Weight (kg)	69 ± 15	70 ± 20	0.911
BMI (kg/m^2^)	23.2 ± 4.0	22.9 ± 9.1	0.843
Waist/hip ratio	0.8 ± 0.1	0.7 ± 0.1	0.423
Heart rate (bpm)	65 ± 7	67 ± 7	0.448
SBP (mmHg)	116 ± 11	114 ± 12	0.521
DBP (mmHg)	72 ± 5	69 ± 10	0.289
MAP (mmHg)	85 ± 7	83 ± 12	0.539
O_2_ sat (%)	98 ± 1	99 ± 1	0.151
E-cigarette usage (years)	3.5 ± 1.7	0 ± 0	**<0.001**
E-cigarette frequency (days/month)	25 ± 5	0 ± 0	**<0.001**
Nicotine (ng/mL)	3.8 ± 4.7	0.0 ± 0.0	**0.009**
Cotinine (ng/mL)	156.3 ± 192.6	0.0 ± 0.0	**0.007**
TC (mg/dL)	136 ± 73	121 ± 79	0.554
HDL (mg/dL)	54 ± 14	40 ± 25	0.065
LDL (mg/dL)	92 ± 32	64 ± 51	0.107
TRIG (mg/dL)	104 ± 61	88 ± 92	0.608
GLU (mg/dL)	90 ± 9	91 ± 7	0.878
HbA_1C_ (%)	5.1 ± 1.1	5.2 ± 0.4	0.356
hs-CRP (mg/L)	1.9 ± 3.8	2.1 ± 3.7	0.740
Estradiol (pg/mL)	72 ± 27	62 ± 18	0.755

Values are mean ± standard deviation (SD). Boldfaced value indicates statistical significance

*M* male, *F* female, *BMI* body mass index, *SBP* systolic blood pressure, *DBP* diastolic blood pressure, *MAP* mean arterial blood pressure, *O*_*2*_* sat* oxygen saturation, *TC* total cholesterol, *HDL* high-density lipoproteins, *LDL* low-density lipoproteins, *TRIG* triglycerides, *GLU* glucose, *HbA1c* hemoglobin A1c, *hs-CRP* high sensitivity C-reactive protein

### Microvascular function

Data for users of e-cigarettes (ECU) and non-users (NU) for red blood flux (RBF) and cutaneous vascular conductance (CVC) is presented in Table 2. Baseline flux and conductance were similar (*p* ≥ 0.098) between groups for all completed reactivity tests. Participants from both groups also showed similar Brownian movement of macromolecules in the cutaneous interstitial space (B_0_, ECU: 14 ± 2 PU vs. NU: 18 ± 21 PU, *p* = 0.107). In addition, no differences (*p* = 0.673) in skin resistance were identified between participants from both groups (ECU: 2.9 ± 0.2 Ω vs. NU: 2.7 ± 0.2 Ω).

**Table 2:** Microvascular function in young users of e-cigarettes and non-users

Variable	Users of e-cigarettes	Non-users	*p* value
Local thermal hyperemia			
Baseline_LTH_ (PU)	31 ± 1	34 ± 2	0.098
RBF_LTH_ (PU)	144 ± 7	167 ± 5	**0.010**
Area_LTH_ (PU s^−1^)	183,501 ± 12,110	221,238 ± 10,244	**0.022**
Baseline_LTH_ (PU/mmHg)	0.33 ± 0.02	0.38 ± 0.03	0.147
CVC_LTH_ (PU/mmHg)	1.73 ± 0.09	1.98 ± 0.07	**0.046**
TTP_LTH_ (s)	976 ± 60	1030 ± 78	0.726
CVC_max_ (PU/mmHg)	2.3 ± 0.1	2.6 ± 0.1	**0.023**
Post-occlusive reactive hyperemia			
Baseline_PORH_ (PU)	34 ± 1	38 ± 2	0.117
RBF_PORH_ (PU)	62 ± 3	76 ± 3	**0.003**
Area_PORH_ (PU s^−1^)	11,913 ± 523	15,459 ± 1412	**0.023**
Baseline_PORH_ (PU/mmHg)	0.41 ± 0.01	0.43 ± 0.02	0.109
CVC_PORH_ (PU/mmHg)	0.74 ± 0.03	0.89 ± 0.05	**0.016**
TTP_PORH_ (s)	15 ± 1	15 ± 1	0.868
CVC_PORH_ (%_max_)	57 ± 13	69 ± 4	**0.004**
Iontophoresis acetylcholine			
Baseline_ACH_ (PU)	31 ± 2	37 ± 3	0.097
RBF_ACH_ (PU)	91 ± 6	114 ± 9	**0.044**
Area_ACH_ (PU s^−1^)	3,420 ± 371	2,898 ± 390	0.241
Baseline_ACH_ (PU/mmHg)	0.37 ± 0.02	0.39 ± 0.03	0.116
CVC_ACH_ (PU/mmHg)	1.01 ± 0.07	1.24 ± 0.07	**0.037**
TTP_ACH_ (s)	70 ± 8	63 ± 7	0.512
CVC_EDD_ (%_max_)	32 ± 3	41 ± 3	**0.034**
Iontophoresis sodium nitroprusside			
Baseline_SNP_ (PU)	32 ± 2	37 ± 4	0.104
RBF_SNP_ (PU)	82 ± 10	68 ± 9	0.328
Area_SNP_ (PU s^−1^)	4,660 ± 475	4,363 ± 331	0.603
Baseline_SNP_ (PU/mmHg)	0.39 ± 0.03	0.41 ± 0.04	0.135
CVC_SNP_ (PU/mmHg)	0.93 ± 0.10	0.77 ± 0.10	0.248
TTP_SNP_ (s)	78 ± 8	66 ± 9	0.333
CVC_EID_ (%_max_)	47 ± 4	35 ± 4	**0.036**
Control			
Baseline_C_ (PU)	39 ± 2	40 ± 3	0.173
RBF_C_ (PU)	38 ± 5	39 ± 3	0.253
Baseline_C_ (PU/mmHg)	0.38 ± 0.02	0.41 ± 0.05	0.198
CVC_C_ (PU/mmHg)	0.45 ± 0.06	0.49 ± 0.07	0.969

Values are mean ± standard error of mean (SEM). Boldfaced value indicates statistical significance. For each test, cutaneous blood flow as red blood cell flux (RBF) was expressed perfusion units (PU) and cutaneous vascular conductance (CVC; CVC = RBF/MAP) was expressed in perfusion units per millimeters of mercury (PU/mmHg)

*LTH* local thermal hyperemia, *PORH* post-occlusive reactive hyperemia, *ACh* acetylcholine, *SNP* sodium nitroprusside, *TTP* time to peak, *PU* perfusion units, *EDD* endothelial-dependent dilation, *EID* endothelial-independent dilation

#### Local thermal hyperemia

**Fig. 1:**
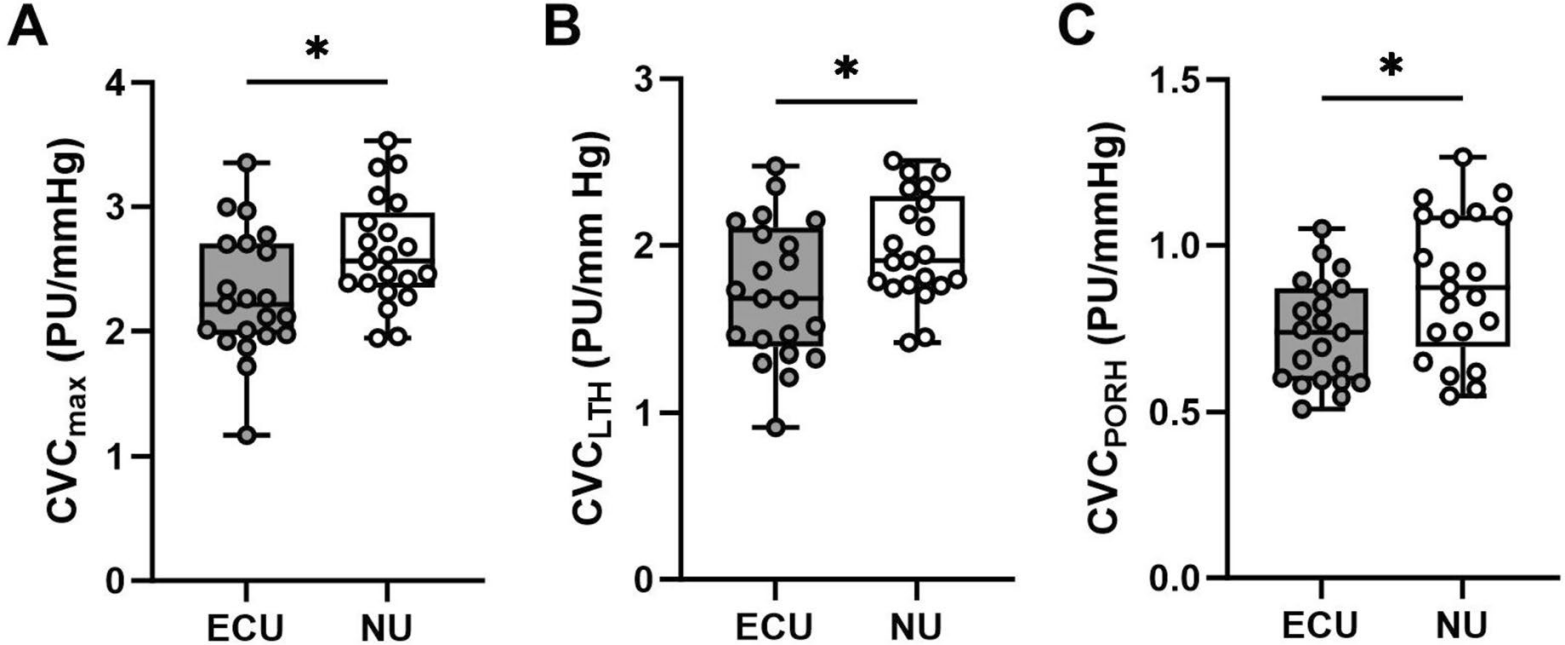
Microvascular function in regular users of E-cigarettes (ECU) and Non-users (NU). Individual data illustrated as Box-and-Whisker plots with minimum and maximum values for **A** overall cutaneous vascular conductance response (CVC_max_), **B** response to local thermal hyperemia (CVC_LTH_), and **C** response to post occlusive reactive hyperemia (CVC_PORH_). Group differences were determined by independent group *t* tests and denoted by * when *p* < 0.05 in users of E-Cigarettes (ECU) vs. Non-Users (NU). CVC: Cutaneous Vascular Conductance; PU: perfusion units

The overall microvascular response to local thermal hyperemia was different between the users of e-cigarettes and non-users. During the initial peak in response to the thermal provocation, the ECU group exhibited a higher CVC response than the non-users (RBF, ECU: 159 ± 8 PU vs. NU: 148 ± 9 PU, *p* = 0.373; CVC, ECU: 1.9 ± 0.1 PU/mmHg vs. NU: 1.4 ± 0.1 PU/mmHg, *p* = 0.013). During the longer phase of the thermal challenge, the ECU group achieved a significantly lower maximal response than the NU for both RBF (ECU: 190 ± 8 PU vs. NU: 222 ± 7 PU, *p* = 0.004) and CVC (ECU: 2.3 ± 0.1 PU/mmHg vs. NU: 2.6 ± 0.1 PU/mmHg, *p* = 0.023, Fig. 1A). Similarly, a significantly (*p* = 0.022) lower response was observed when considering the area under the curve in response to the thermal challenge. In addition, the overall plateau response to the thermal challenge was significantly lower in the ECU group than in the NU (RBF: *p* = 0.010, Cohen’s *d* = 0.71*;* CVC, *p* = 0.029, Cohen’s *d* = 0.62, Fig. 1B).

#### Post-occlusive reactive hyperemia

For the post-occlusive reactive hyperemia test, the users of e-cigarettes showed a significantly lower overall hyperemic response compared to the non-users (RBF: *p* = 0.003, Cohen’s *d* = 1.07; CVC: *p* = 0.016, Cohen’s *d* = 0.94, Fig. 1C). In addition, the maximal hyperemic response was also significantly lower in the ECU when compared to the NU group for RBF (ECU: 134 ± 8 PU vs. NU: 161 ± 6 PU, *p* = 0.008) and CVC (ECU: 1.6 ± 0.1 PU/mmHg vs. NU: 1.9 ± 0.1 PU/mmHg, *p* = 0.003). Even when considering the area under the curve in response to this test, users of e-cigarettes showed a significantly (*p* = 0.023) lower response than the non-users. Overall, the response to the post-occlusive reactive hyperemia test was proportionally lower in the users of e-cigarettes than in the non-users when compared to the maximal response (ECU: 57 ± 3% maximal dilation vs. NU: 69 ± 4% maximal dilation, *p* = 0.044).

#### Endothelial-dependent dilation

**Fig. 2:**
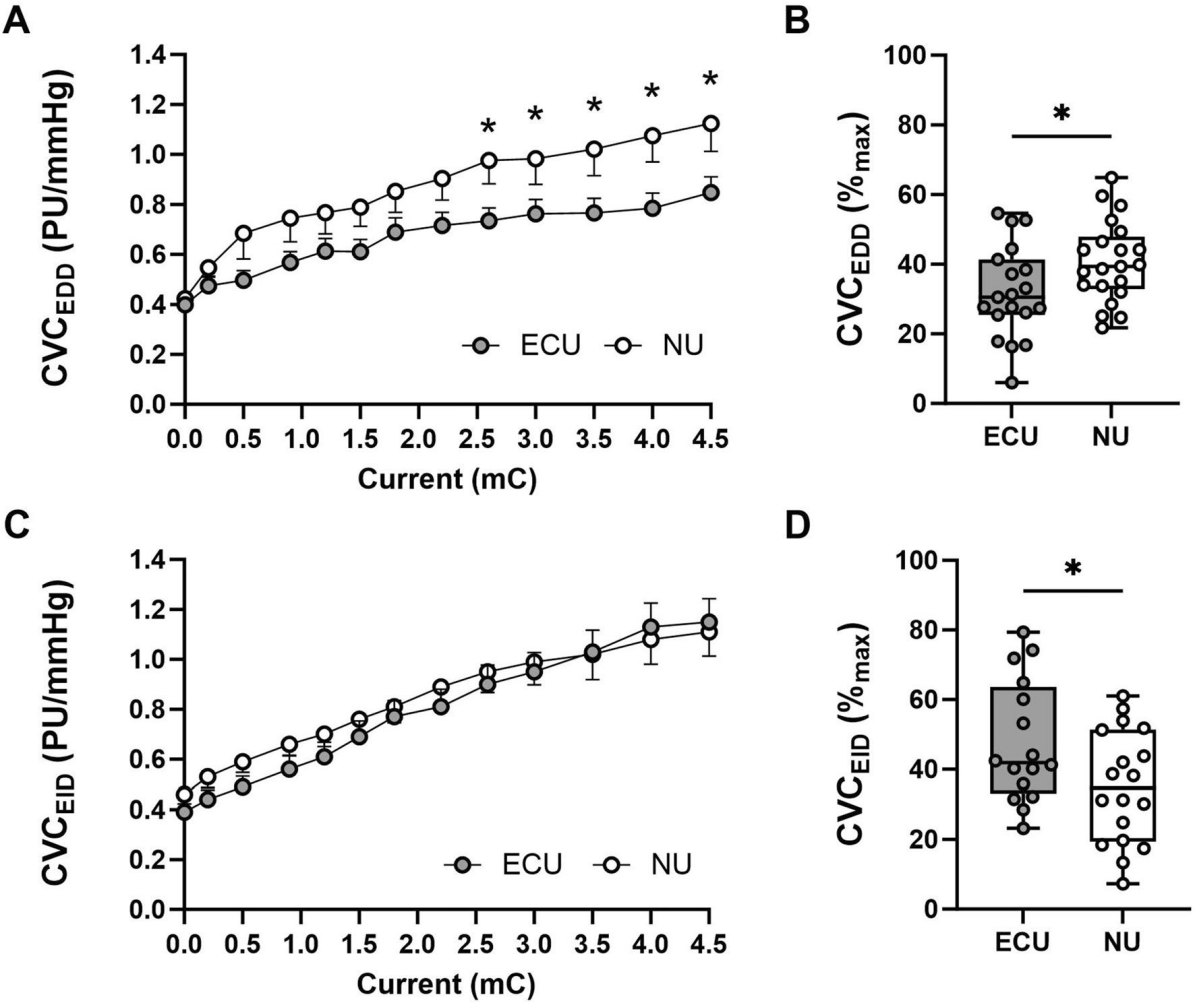
Endothelial-Dependent (EDD) and Endothelial-Independent (EID) dilation in regular users of E-cigarettes (ECU) and Non-users (NU). Endothelial Dependent (EDD) and Endothelial Independent (EID) dilation in users of E-cigarettes (ECU) and Non-Users (NU). Individual data is illustrated as Box-and-Whisker plots with minimum and maximum values as well as dose response based on an incremental current. **A** Dose response to acetylcholine; **B** Maximal endothelial-dependent dilation; **C** Dose response to sodium nitroprusside; **D** Maximal endothelial-independent dilation. Differences were determined by independent group *t* tests and denoted by * when *p* < 0.05 in users of E-Cigarettes vs. Non-Users. *CVC* cutaneous vascular conductance, *PU* perfusion units

During the endothelium-dependent dilation test, both groups showed similar (*p* ≥ 0.064) responses to the delivery of acetylcholine at a lower charge (0.2–2.3 mC). However, the ECU group showed a significantly (*p* ≤ 0.043) reduced response rate at charges equal to or greater than 2.5 mC when compared to the NU group (Fig. 2A**)**. Specifically, the response to the iontophoresis of acetylcholine at the highest cumulative charge (4.5 mC) was significantly lower in the e-cigarette group than in the non-users for both RBF (ECU: 72 ± 5 PU vs. NU: 98 ± 9 PU, *p* = 0.012, Cohen’s *d* = 1.15) and CVC (ECU: 0.8 ± 0.1 PU/mmHg vs. NU: 1.1 ± 0.1 PU/mmHg, *p* = 0.034, Cohen’s *d* = 1.01). In addition, the response to this reactivity test was proportionally lower in the users of e-cigarettes than in the no users when compared to the maximal response (ECU: 32 ± 3% maximal dilation vs. NU: 41 ± 3% maximal dilation, *p* = 0.034; Fig. 2B).

#### Endothelial-independent dilation

During iontophoresis with SNP, no differences (*p* ≥ 0.092) were observed in the overall or maximal response between both groups at any charge (Fig. 2C**)**. The response observed at 4.5 mC was similar for both groups both at RBF (ECU: 101 ± 10 PU vs. NU: 95 ± 9 PU, *p* = 0.622, Cohen’s *d* = 1.01) and CVC (ECU: 1.1 ± 0.1 PU/mmHg vs. NU: 1.1 ± 0.1 PU/mmHg, *p* = 0.773, Cohen’s *d* = 0.89). However, when considering the maximal dilatory capacity, the ECU group showed an overall higher proportional response to SNP than the NU group (ECU: 47 ± 4% maximal dilation vs. NU: 35 ± 4% maximal dilation, *p* = 0.036, Fig. 2D).

#### Control site

Non-specific vasodilation was monitored in a control site. No differences (*p* ≥ 0.114) were identified between either group at any point during the thirty-minute monitoring period. The non-specific response observed was similar between both groups when considering maximal response (ECU: 16 ± 2% maximal dilation vs. NU: 16 ± 2% maximal dilation, *p* = 0.781).

### Macrovascular function

Table 3 presents vascular function in the brachial artery in users of e-cigarettes and non-users. The baseline diameter was similar (*p* = 0.336) between groups. The FMD response was similar (*p* = 0.086; Cohen’s *d* = 0.70) between both groups. No differences (*p* = 0.091; Cohen’s *d* = 0.96) in FMD normalized for shear rate were identified. In addition, the cumulative response when controlling for low flow-mediated constriction was similar (*p* = 0.068) in both groups.

**Table 3:** Macrovascular function in young users of e-cigarettes and non-users

Variable	Users e-cigarettes	Non-users	*p* value
Baseline diameter (mm)	3.47 ± 0.10	3.31 ± 0.12	0.336
Peak diameter (mm)	3.77 ± 0.12	3.67 ± 0.13	0.541
FMD (%)	8.5 ± 0.8	10.7 ± 0.9	0.086
L-FMC (%)	−1.8 ± 0.7	−1.9 ± 0.8	0.864
c-FMD (%)	10.1 ± 0.8	12.7 ± 1.1	0.068
Shear (s^−1^, AUC)	52,075 ± 4610	44,781 ± 4041	0.490
FMD (%)/shear (s^−1^, AUC)	0.282 ± 0.04	0.360 ± 0.03	0.091
Time to peak (s)	52 ± 17	35 ± 76	0.327

Values are mean ± standard error of mean (SEM)

*FMD* flow-mediated dilation, *L-FMC* low flow-mediated constriction, *c-FMD* composite FMD, *AUC* area under the curve

### Relationship between E-cigarette usage and vascular health

**Fig. 3:**
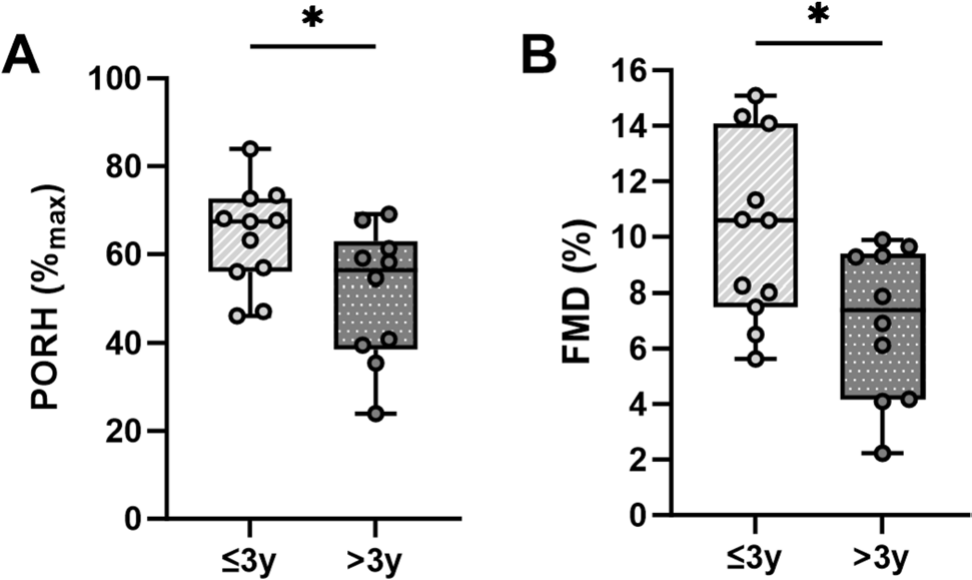
Micro (**A**) and Macro (**B**) Vascular Function in Young Users of E-Cigarettes Based on Length of Usage. Differences in **A** microvascular function assessed through post-occlusive reactive hyperemia and **B** macrovascular function assessed through flow-mediated dilation in users of e-cigarettes for shorter than three years (≤ 3 year) and for longer than three years (> 3 year). Differences were determined by independent group *t* tests and denoted by * when *p* < 0.05 in > 3 year vs. ≤ 3 year of usage

A secondary analysis was completed to evaluate the potential relationship between e-cigarette usage and vascular health assessments. Table 4 and Fig. 3 summarize micro and macrovascular results based on length of e-cigarette usage. Significant negative associations have been identified between years of e-cigarette usage and the microvascular hyperemic response (*r* = −0.421; *p* = 0.001). Indeed, the PORH response was significantly (*p* ≤ 0.044) lower in those individuals that used e-cigarettes for longer than three years when compared to shorter length of usage (> 3 year: 51 ± 5%_max_ vs. ≤ 3 year: 64 ± 3%_max_; *p* = 0.039, Fig. 3A). Those that used e-cigarettes for longer than 3 years also exhibited a significantly (*p* ≤ 0.044) lower maximal hyperemic response both in RBF (> 3 year: 56 ± 3 PU vs. ≤3 year: 68 ± 4 PU, *p* = 0.044) and CVC (> 3 year: 0.70 ± 0.03 PU/mmHg vs. ≤ 3 year: 0.80 ± 0.04 PU, *p* = 0.036). To note, differences were independent of nicotine usage, and only moderate associations between cotinine concentrations and endothelial-independent response (*r* = 0.326; *p* = 0.031) have been observed. On the other hand, no relationships have been observed between length of usage or frequency of usage and maximal dilation, endothelial-dependent and endothelial-independent mechanisms. We also evaluated the response of larger vessels and identified that macrovascular health was negatively associated with cotinine levels (*r* = −0.490; *p* = 0.002), frequency of usage (*r* = −0.537; *p* = 0.015), and length of usage (*r* = −0.309; *p* = 0.049). Indeed, those individuals that used e-cigarettes for longer than 3 years present a significantly lower macrovascular response than those users for a shorter time (> 3 year: 6.9 ± 0.9% vs. ≤ 3 year: 10.1 ± 1.0%, *p* = 0.002, Fig. 3B). To note, no differences (*p* ≥ 0.236) in demographics, or clinical laboratory values were identified between individuals that used e-cigarettes for longer or shorter than 3 years and both nicotine and cotinine concentrations were similar (*p* ≥ 0.312) between both groups.

**Table 4:** Micro- and macro-vascular function in young users of e-cigarettes based on length of usage

Variable	≤3 years	>3 years	*p* value
Microvascular function			
Local thermal hyperemia			
Baseline_LTH_ (PU)	30 ± 2	30 ± 2	0.627
RBF_LTH_ (PU)	143 ± 6	148 ± 5	0.726
Area_LTH_ (PU s^−1^)	179,851 ± 12,328	196,824 ± 14,652	0.414
Baseline_LTH_ (PU/mmHg)	0.33 ± 0.02	0.35 ± 0.03	0.846
CVC_LTH_ (PU/mmHg)	1.70 ± 0.12	1.78 ± 0.15	0.683
TTP_LTH_ (s)	885 ± 98	1066 ± 73	0.156
Post-occlusive reactive hyperemia			
Baseline_PORH_ (PU)	34 ± 1	34 ± 2	0.608
RBF_PORH_ (PU)	68 ± 4	56 ± 3	**0.044**
Area_PORH_ (PU s^−1^)	12,649 ± 891	11,071 ± 779	0.058
Baseline_PORH_ (PU/mmHg)	0.41 ± 0.01	0.41 ± 0.01	0.809
CVC_PORH_ (PU/mmHg)	0.80 ± 0.04	0.70 ± 0.03	**0.036**
TTP_PORH_ (s)	17 ± 2	14 ± 1	0.250
Iontophoresis with acetylcholine			
Baseline_ACH_ (PU)	29 ± 2	34 ± 3	0.712
RBF_ACH_ (PU)	91 ± 10	92 ± 8	0.904
Area_ACH_ (PU s^−1^)	3175 ± 414	3403 ± 336	0.563
Baseline_ACH_ (PU/mmHg)	0.35 ± 0.02	0.39 ± 0.04	0.131
CVC_ACH_ (PU/mmHg)	0.99 ± 0.11	1.05 ± 0.07	0.698
TTP_ACH_ (s)	78 ± 10	64 ± 7	0.371
Iontophoresis with sodium nitroprusside			
Baseline_SNP_ (PU)	31 ± 2	34 ± 6	0.436
RBF_SNP_ (PU)	69 ± 10	96 ± 12	0.166
Area_SNP_ (PU s^−1^)	4135 ± 494	5250 ± 828	0.253
Baseline_SNP_ (PU/mmHg)	0.37 ± 0.03	0.41 ± 0.04	0.425
CVC_SNP_ (PU/mmHg)	0.85 ± 0.11	1.04 ± 0.17	0.349
TTP_SNP_ (s)	70 ± 10	89 ± 7	0.273
Control			
Baseline_C_ (PU)	39 ± 2	39 ± 3	0.515
RBF_C_ (PU)	37 ± 6	42 ± 3	0.787
Baseline_C_ (PU/mmHg)	0.37 ± 0.01	0.38 ± 0.02	0.634
CVC_C_ (PU/mmHg)	0.44 ± 0.09	0.45 ± 0.06	0.884
Macrovascular function			
Baseline diameter (mm)	3.49 ± 0.16	3.45 ± 0.17	0.812
Peak diameter (mm)	3.85 ± 0.17	3.69 ± 0.17	0.509
FMD (%)	10.1 ± 1.0	6.9 ± 0.9	**0.002**
L-FMC (%)	−1.5 ± 0.8	−2.1 ± 1.1	0.685
c-FMD (%)	11.2 ± 1.4	9.1 ± 0.7	0.090
Shear (s^−1^, AUC)	51,903 ± 6193	48,862 ± 7349	0.755
FMD (%)/shear (s^−1^, AUC)	0.326 ± 0.06	0.294 ± 0.06	0.366
Time to peak (s)	50 ± 3	54 ± 7	0.554

Values are mean ± standard error of mean (SEM). Boldfaced value indicates statistical significance. For each test, cutaneous blood flow as red blood cell flux (RBF) was expressed in perfusion units (PU) and cutaneous vascular conductance (CVC; CVC = RBF/MAP) was expressed in perfusion units per millimeters of mercury (PU/mmHg)

*LTH* local thermal hyperemia, *PORH* post-occlusive reactive hyperemia, *ACh* acetylcholine, *SNP* sodium nitroprusside, *TTP* time to peak, *PU* perfusion units, *FMD* flow-mediated dilation, *L-FMC* low flow-mediated constriction, *c-FMD* composite FMD, *AUC* area under the curve

## Discussion

Impaired vascular health is a predictive marker of future cardiovascular dysfunction. Early evidence supports that the use of e-cigarettes may damage the vasculature and increase the risk of CVD development. The present investigation has expanded prevailing results and identified the presence of premature vascular dysfunction in young and apparently healthy regular users of e-cigarettes, specifically within the microvasculature. An interesting finding is that vascular damage in smaller blood vessels preceded damage in larger blood vessels in those that have been regularly using e-cigarettes for longer than three years. The findings of this investigation also identified that different molecular mechanisms are involved in the diminished vascular response observed in users of e-cigarettes including both endothelial-dependent and independent mechanism. Thus, the present study provides novel information on the link between e-cigarette usage and premature vascular damage and expands current knowledge related to a use timeline.

### E-cigarette usage and vascular health

Currently, e-cigarettes are primarily used by young individuals who have never smoked combustible tobacco but frequently use these newer and more attractive products [36]. With the rapid increase in usage, there are rising concerns regarding the impact of the consumption of these products on users’ health. Recent studies have provided information related to the effects of these products on the health of adult users supporting that a single exposure to e-cigarettes caused a reduction in either micro [37, 38] or macro [8, 10, 11, 39] vascular function. Our results expand previous findings and identify that young regular users of e-cigarettes exhibit reduced vascular function primarily in the microvasculature, while the response of larger vessels still appears apparently preserved when length of use was not considered. It is important to note that the functionality of the small vessels plays a pivotal role in cardiovascular health and their dysfunction is considered one the earliest indicators of cardiovascular disease risk [17, 18]. Indeed, alterations in the microvasculature often precede dysfunctions observed in larger vessels [40] and are commonly considered a better predictor of long-term outcomes and adverse cardiovascular events [41–43]. Similar to our findings, studies completed in animal models exposed to e-cigarettes also described damage in smaller arteries prior to larger ones [44]. Thus, our data identifies, for the first time, that young users of e-cigarettes exhibit premature microvascular dysfunction when compared to demographically matched non-users that may precede damage in larger blood vessels.

Vascular function is governed by different mechanisms, with endothelial function as one of the main ones. The endothelium, a key regulator of vascular tone and blood flow, controls vasodilatory and vasoconstrictive mechanisms by synthesizing mediators such as nitric oxide, a potent vasodilator [45–47]. Reduced bioavailability of this vasodilator is associated with lower endothelial-dependent vasodilation and is considered a marker of poor cardiovascular health [48]. It is well established that traditional combustible tobacco usage is associated with worse endothelial function and lowered nitric oxide levels [49–51]. Preliminary evidence in the microvasculature [19] and other vascular beds [12] supports that e-cigarette use also leads to a reduction of this mediator [12]. Our results align with previous observations and support that regular consumers of e-cigarettes exhibit a reduction of this essential vasodilator also in the microcirculation, as evaluated through LTH. In addition, other critical mediators including prostaglandins and EDHF [52] are also involved in the endothelial-mediated response of the small vessels and may be also impaired in those that consume e-cigarettes, as suggested by our data from the PORH, LTH and iontophoresis with ACh reactivity tests. Our results support reduced endothelial-dependent microvascular function which was described in another cohort of users of e-cigarettes [19]. Recently, a study completed in a preclinical model exposed to e-cigarette vapor has also identified impaired endothelial-independent mechanisms [53]. In our study, endothelial-independent vasodilation, evaluated through iontophoresis with SNP, was similar between the groups or even higher (when percentage of maximal dilation was evaluated) in the ECU group, suggesting intact vascular smooth muscle function and even a potential overcompensation for impaired endothelial-dependent function in those young adults that are regular users of e-cigarettes. While nicotine has the ability to dimmish endothelium-independent vasodilation via interaction with ATP-sensitive K^+^ channels located on the vascular smooth muscle [54], studies evaluating the functionality of smooth muscle in users of cigarettes have mixed results showing reduced [55–57] or unchanged [58] endothelial-independent vasodilation. It is possible that length of tobacco usage may play a role in the development of general vascular dysfunction, evident first by endothelial impairments and then, by reduced vascular smooth muscle vasodilation. Analyzing the observed microvascular responses, we should also consider the possibility that e-cigarette usage hyperactivates the response initiated by sensory nerves, as evaluated via the initial response to thermal challenge, particularly due to their involvement in the initial response to the thermal challenge [59]. Prevailing data also support that acute exposure to e-cigarettes, as well as to traditional combustible tobacco, increases skin sympathetic nerve activity [60] that could lead to a poor vasodilatory response. In summary, findings from the present study expand current knowledge and demonstrate that young individuals that are regular e-cigarette users present with early alterations in different molecular mechanisms that govern microvascular function, one of the earliest markers of CVD.

### E-cigarette usage length and vascular health

In the present study, we observed preserved macrovascular function in users of e-cigarettes, similar [15] and opposite [12] results have been described. Interestingly our study identified that young adults that have been using e-cigarettes for longer time also present with reduced functionality of larger vessels when compared to more recent users. Particularly, we have observed that flow-induced vasodilation in the micro- and macrovasculature, as evaluated by post-occlusive reactive hyperemia and flow-mediated dilation respectively, was reduced in those who used e-cigarette products for a longer time, while other vasodilatory mechanisms were similar between the subgroups. It is possible that over time, impairment in the microcirculation can increase vascular resistance to flow, resulting in greater retrograde and oscillatory shear in conduit arteries [61], potentially triggering an increase in oxidative stress and associated reduction in the synthesis of vasodilatory mediators [62, 63]. It is also possible that the endothelial glycocalyx, an essential mechanotransductor of shear stress that initiates intracellular signaling to promote vasodilation, will be deteriorated by the prolonged use of e-cigarettes, as previously observed in cigarette smoking [64]. In fact, recent studies have shown that endothelial glycocalyx integrity can be improved after three months of smoking cessation [65] while no changes have been observed in traditional tobacco users that switch to e-cigarettes [66]. Thus, it is possible that prolonged use of e-cigarette damages the endothelial glycocalyx resulting in decreased flow-induced vasodilation in both the micro and macro circulation. Independent of the mechanisms, the present results propose a link between length of usage and vascular damage denoting that the observed deleterious effects are not just associated with nicotine usage, as others have also identified [44, 67, 68]. Indeed, several preclinical studies reported impaired endothelial function following exposure to nicotine-free aerosol [4, 69, 70]. To note, thermal degradation of common e-liquid solvents (propylene glycol or vegetable glycerin) and the subsequent formation of different aldehydes [71, 72] have also been linked to vascular impairments [68]. In addition, early observations have also identified that certain flavor additives (i.e. cinnamon, menthol) may increase oxidative stress [73, 74] and diminish the synthesis of vasodilators in endothelial cells [75].

### Study limitations

Despite the novel findings of the present study, there are also several limitations that should be considered. The study was purposely conducted in young, apparently healthy adults to eliminate the potential negative effects of aging on the vasculature. Physical activity levels, dietary intake, current and previous alcohol and drug use, or sleep patterns were similar between users and non-users. In addition, similar number of participants from both groups self-reported having consumed cannabis ever in the lifetime. However, we cannot conclude that some of these lifestyle choices or other factors not measured could influence the vascular response observed. Another limitation is related to the evaluation of microvascular function using reactivity tests. Despite providing very early insights into cardiovascular health, the technical complexity of the assessments limits the possibility to easily translated this testing into clinical settings, minimizing the ability of identifying early damage in larger and/or other populations. Another important limitation to consider is the rapidly changing market related to e-cigarettes. Indeed, from 2020 to 2022, the total number of e-cigarettes brands on the US market increased from 184 to 269 [76], providing a large variety of flavors and device types, estimated to offer 20,000 different types of e-liquids [77]. Thus, we should consider that the diversity of e-cigarette products might result in unique effects on users’ vascular health that may differ from the present findings.

## Conclusion

In conclusion, results from the present study demonstrate that young and apparently healthy adults that regularly use e-cigarettes exhibit reduced vascular function that initially impacts the microcirculation. Our findings identified that those who have been users of e-cigarettes for a longer time also exhibit reduced functionality of larger blood vessels, independent of nicotine content, denoting the appearance of systemic vascular damage. We have also identified different molecular mechanisms that govern vascular health as impaired in regular users of e-cigarettes representing the multifaceted nature of the observed damage. Results from the present study add to the growing body of literature emphasizing that the usage of e-cigarettes is not a harmless action and could contribute to the onset of premature cardiovascular diseases.

## Data Availability

The data presented in this article will be shared on reasonable request to the corresponding author.

## References

[1] Cooper M, Park-Lee E, Ren C, Cornelius M, Jamal A, Jamal KA (2022). Cullen KA (2022) Notes from the field: e-Cigarette use among middle and high school students—United States. MMWR Morb Mortal Wkly Rep.

[2] Pearson JL, Richardson A, Niaura RS, Vallone DM, Abrams DB (2012). e-Cigarette awareness, use, and harm perceptions in US adults. Am J Public Health.

[3] Olfert IM, DeVallance E, Hoskinson H, Branyan KW, Clayton S, Pitzer CR, Sullivan DP, Breit MJ, Wu Z, Klinkhachorn P, Mandler WK, Erdreich BH, Ducatman BS, Bryner RW, Dasgupta P, Chantler PD (2018). Chronic exposure to electronic cigarettes results in impaired cardiovascular function in mice. J Appl Physiol.

[4] El-Mahdy MA, Mahgoup EM, Ewees MG, Eid MS, Abdelghany TM, Zweier JL (2021). Long-term electronic cigarette exposure induces cardiovascular dysfunction similar to tobacco cigarettes: role of nicotine and exposure duration. Am J Physiol Heart Circ Physiol.

[5] Vlachopoulos C, Ioakeimidis N, Abdelrasoul M, Terentes-Printzios D, Georgakopoulos C, Pietri P, Stefanadis C, Tousoulis D (2016). Electronic cigarette smoking increases aortic stiffness and blood pressure in young smokers. J Am Coll Cardiol.

[6] Benthien J, Meusel M, Cayo Talavera S, Eitel I, Dromann D, Franzen KF (2022). JUULing and heating lead to a worsening of arterial stiffness. Medicines (Basel).

[7] Gonzalez JE, Cooke WH (2021). Acute effects of electronic cigarettes on arterial pressure and peripheral sympathetic activity in young nonsmokers. Am J Physiol Heart Circ Physiol.

[8] Kuntic M, Oelze M, Steven S, Kroller-Schon S, Stamm P, Kalinovic S, Frenis K, Vujacic-Mirski K, Bayo Jimenez MT, Kvandova M, Filippou K, Al Zuabi A, Bruckl V, Hahad O, Daub S, Varveri F, Gori T, Huesmann R, Hoffmann T, Schmidt FP, Keaney JF, Daiber A, Munzel T (2020). Short-term e-cigarette vapour exposure causes vascular oxidative stress and dysfunction: evidence for a close connection to brain damage and a key role of the phagocytic NADPH oxidase (NOX-2). Eur Heart J.

[9] Ben Taleb Z, Dabroy D, Akins J, Nelson MD, Kalan ME, Rezk-Hanna M, Brothers RM (2023). Pod-based e-cigarettes versus combustible cigarettes: the impact on peripheral and cerebral vascular function and subjective experiences. Tob Induc Dis.

[10] Chatterjee S, Caporale A, Tao JQ, Guo W, Johncola A, Strasser AA, Leone FT, Langham MC, Wehrli FW (2021). Acute e-cig inhalation impacts vascular health: a study in smoking naive subjects. Am J Physiol Heart Circ Physiol.

[11] Biondi-Zoccai G, Sciarretta S, Bullen C, Nocella C, Violi F, Loffredo L, Pignatelli P, Perri L, Peruzzi M, Marullo AGM, De Falco E, Chimenti I, Cammisotto V, Valenti V, Coluzzi F, Cavarretta E, Carrizzo A, Prati F, Carnevale R, Frati G (2019). Acute effects of Heat-Not-Burn, electronic Vaping, and traditional tobacco combustion cigarettes: the Sapienza University of Rome-Vascular assessment of proatherosclerotic effects of smoking (SUR-VAPES) 2 randomized trial. J Am Heart Assoc.

[12] Mohammadi L, Han DD, Xu F, Huang A, Derakhshandeh R, Rao P, Whitlatch A, Cheng J, Keith RJ, Hamburg NM, Ganz P, Hellman J, Schick SF, Springer ML (2022). Chronic e-cigarette use impairs endothelial function on the physiological and cellular levels. Arterioscler Thromb Vasc Biol.

[13] Farsalinos KE, Tsiapras D, Kyrzopoulos S, Savvopoulou M, Voudris V (2014). Acute effects of using an electronic nicotine-delivery device (electronic cigarette) on myocardial function: comparison with the effects of regular cigarettes. BMC Cardiovasc Disord.

[14] Szoltysek-Boldys I, Sobczak A, Zielinska-Danch W, Barton A, Koszowski B, Kosmider L (2014). Influence of inhaled nicotine source on arterial stiffness. Przegl Lek.

[15] Boakye E, Uddin SMI, Osuji N, Meinert J, Obisesan OH, Mirbolouk M, Tasdighi E, El-Shahawy O, Erhabor J, Osei AD, Rajan T, Patatanian M, Holbrook JT, Bhatnagar A, Biswal SS, Blaha MJ (2023). Examining the association of habitual e-cigarette use with inflammation and endothelial dysfunction in young adults: the VAPORS-Endothelial function study. Tob Induc Dis.

[16] Masi S, Rizzoni D, Taddei S, Widmer RJ, Montezano AC, Luscher TF, Schiffrin EL, Touyz RM, Paneni F, Lerman A, Lanza GA, Virdis A (2021). Assessment and pathophysiology of microvascular disease: recent progress and clinical implications. Eur Heart J.

[17] Kenney WL (2017). Edward F. Adolph distinguished lecture: skin-deep insights into vascular aging. J Appl Physiol.

[18] Kruger A, Stewart J, Sahityani R, O'Riordan E, Thompson C, Adler S, Garrick R, Vallance P, Goligorsky MS (2006). Laser Doppler flowmetry detection of endothelial dysfunction in end-stage renal disease patients: correlation with cardiovascular risk. Kidney Int.

[19] Halstead KM, Wetzel EM, Cho JL, Stanhewicz AE (2023). Sex differences in oxidative stress-mediated reductions in microvascular endothelial function in young adult e-cigarette users. Hypertension.

[20] Morean ME, Krishnan-Sarin S, Sussman S, Foulds J, Fishbein H, Grana R, O'Malley SS (2019). Psychometric evaluation of the e-cigarette dependence scale. Nicotine Tob Res.

[21] Foulds J, Veldheer S, Yingst J, Hrabovsky S, Wilson SJ, Nichols TT, Eissenberg T (2015). Development of a questionnaire for assessing dependence on electronic cigarettes among a large sample of ex-smoking e-cigarette users. Nicotine Tob Res.

[22] Roustit M, Cracowski JL (2013). Assessment of endothelial and neurovascular function in human skin microcirculation. Trends Pharmacol Sci.

[23] Minson CT (2010). Thermal provocation to evaluate microvascular reactivity in human skin. J Appl Physiol.

[24] Brunt VE, Minson CT (2012). KCa channels and epoxyeicosatrienoic acids: major contributors to thermal hyperaemia in human skin. J Physiol.

[25] Lorenzo S, Minson CT (2007). Human cutaneous reactive hyperaemia: role of BKCa channels and sensory nerves. J Physiol.

[26] Cracowski JL, Minson CT, Salvat-Melis M, Halliwill JR (2006). Methodological issues in the assessment of skin microvascular endothelial function in humans. Trends Pharmacol Sci.

[27] Durand S, Tartas M, Bouye P, Koitka A, Saumet JL, Abraham P (2004). Prostaglandins participate in the late phase of the vascular response to acetylcholine iontophoresis in humans. J Physiol.

[28] Droog EJ, Henricson J, Nilsson GE, Sjoberg F (2004). A protocol for iontophoresis of acetylcholine and sodium nitroprusside that minimises nonspecific vasodilatory effects. Microvasc Res.

[29] Blaise S, Hellmann M, Roustit M, Isnard S, Cracowski JL (2010). Oral sildenafil increases skin hyperaemia induced by iontophoresis of sodium nitroprusside in healthy volunteers. Br J Pharmacol.

[30] Pienaar PR, Micklesfield LK, Gill JM, Shore AC, Gooding KM, Levitt NS, Lambert EV (2014). Ethnic differences in microvascular function in apparently healthy South African men and women. Exp Physiol.

[31] Rodriguez-Miguelez P, Seigler N, Harris RA (2016). Ultrasound assessment of endothelial function: a technical guideline of the flow-mediated dilation test. J Vis Exp.

[32] Harris RA, Nishiyama SK, Wray DW, Richardson RS (2010). Ultrasound assessment of flow-mediated dilation. Hypertension.

[33] Gori T, Dragoni S, Lisi M, Di Stolfo G, Sonnati S, Fineschi M, Parker JD (2008). Conduit artery constriction mediated by low flow a novel noninvasive method for the assessment of vascular function. J Am Coll Cardiol.

[34] Lakens D (2013). Calculating and reporting effect sizes to facilitate cumulative science: a practical primer for t-tests and ANOVAs. Front Psychol.

[35] Cohen J (1973). Eta-squared and partial eta-squared in fixed factor ANOVA designs. Educ Psychol Measur.

[36] Sanford BT, Brownstein NC, Baker NL, Palmer AM, Smith TT, Rojewski AM, Toll BA (2023). Shift from smoking cigarettes to vaping nicotine in young adults. JAMA Intern Med.

[37] Lyytinen G, Brynedal A, Anesater E, Antoniewicz L, Blomberg A, Wallen H, Bosson JA, Hedman L, Mobarrez F, Tehrani S, Lundback M (2023). Electronic cigarette vaping with nicotine causes increased thrombogenicity and impaired microvascular function in healthy volunteers: a randomised clinical trial. Cardiovasc Toxicol.

[38] Chaumont M, de Becker B, Zaher W, Culie A, Deprez G, Melot C, Reye F, Van Antwerpen P, Delporte C, Debbas N, Boudjeltia KZ, van de Borne P (2018). Differential effects of e-cigarette on microvascular endothelial function, arterial stiffness and oxidative stress: a randomized crossover trial. Sci Rep.

[39] Carnevale R, Sciarretta S, Violi F, Nocella C, Loffredo L, Perri L, Peruzzi M, Marullo AG, De Falco E, Chimenti I, Valenti V, Biondi-Zoccai G, Frati G (2016). Acute impact of tobacco vs electronic cigarette smoking on oxidative stress and vascular function. Chest.

[40] Anderson TJ, Charbonneau F, Title LM, Buithieu J, Rose MS, Conradson H, Hildebrand K, Fung M, Verma S, Lonn EM (2011). Microvascular function predicts cardiovascular events in primary prevention: long-term results from the Firefighters and Their Endothelium (FATE) study. Circulation.

[41] Rossi M, Matteucci E, Pesce M, Consani C, Franzoni F, Santoro G, Giampietro O (2013). Peripheral microvascular dysfunction as an independent predictor of atherosclerotic damage in type 1 diabetes patients: a preliminary study. Clin Hemorheol Microcirc.

[42] Matsuda J, Murai T, Kanaji Y, Usui E, Araki M, Niida T, Ichijyo S, Hamaya R, Lee T, Yonetsu T, Isobe M, Kakuta T (2016). Prevalence and clinical significance of discordant changes in fractional and coronary flow reserve after elective percutaneous coronary intervention. J Am Heart Assoc.

[43] Mohammed AQ, Abdu FA, Su Y, Liu L, Yin G, Feng Y, Zhang W, Xu Y, Xu D, Che W (2023). Prognostic significance of coronary microvascular dysfunction in patients with heart failure with preserved ejection fraction. Can J Cardiol.

[44] Mills A, Frazier J, Plants R, Burrage E, Coblentz T, Nassabeh S, Robinson M, Chantler PD, Olfert IM (2023). Effects of electronic cigarette E-liquid and device wattage on vascular function. Toxicol Appl Pharmacol.

[45] Ignarro LJ (2002). Nitric oxide as a unique signaling molecule in the vascular system: a historical overview. J Physiol Pharmacol.

[46] Galley HF, Webster NR (2004). Physiology of the endothelium. Br J Anaesth.

[47] Tousoulis D, Kampoli AM, Tentolouris C, Papageorgiou N, Stefanadis C (2012). The role of nitric oxide on endothelial function. Curr Vasc Pharmacol.

[48] Napoli C, Ignarro LJ (2009). Nitric oxide and pathogenic mechanisms involved in the development of vascular diseases. Arch Pharm Res.

[49] Michael Pittilo R (2000). Cigarette smoking, endothelial injury and cardiovascular disease. Int J Exp Pathol.

[50] Barua RS, Ambrose JA, Eales-Reynolds LJ, DeVoe MC, Zervas JG, Saha DC (2001). Dysfunctional endothelial nitric oxide biosynthesis in healthy smokers with impaired endothelium-dependent vasodilatation. Circulation.

[51] Kugiyama K, Yasue H, Ohgushi M, Motoyama T, Kawano H, Inobe Y, Hirashima O, Sugiyama S (1996). Deficiency in nitric oxide bioactivity in epicardial coronary arteries of cigarette smokers. J Am Coll Cardiol.

[52] Cracowski JL, Roustit M (2016). Current methods to assess human cutaneous blood flow: an updated focus on laser-based-techniques. Microcirculation.

[53] El-Mahdy MA, Ewees MG, Eid MS, Mahgoup EM, Khaleel SA, Zweier JL (2022). Electronic cigarette exposure causes vascular endothelial dysfunction due to NADPH oxidase activation and eNOS uncoupling. Am J Physiol Heart Circ Physiol.

[54] Mayhan WG, Sharpe GM (2002). Acute and chronic treatment with nicotine impairs reactivity of arterioles in response to activation of potassium channels. J Cardiovasc Pharmacol.

[55] Edvinsson ML, Andersson SE, Xu CB, Edvinsson L (2008). Cigarette smoking leads to reduced relaxant responses of the cutaneous microcirculation. Vasc Health Risk Manag.

[56] Lind L, Sarabi M, Millgard J, Kahan T (2002). Endothelium-dependent vasodilation is impaired in apparently healthy subjects with a family history of myocardial infarction. J Cardiovasc Risk.

[57] Lanza GA, Spera FR, Villano A, Russo G, Di Franco A, Lamendola P, Crea F (2015). Effect of smoking on endothelium-independent vasodilatation. Atherosclerosis.

[58] Ozaki K, Hori T, Ishibashi T, Nishio M, Aizawa Y (2010). Effects of chronic cigarette smoking on endothelial function in young men. J Cardiol.

[59] Brunt VE, Minson CT (2011). Cutaneous thermal hyperemia: more than skin deep. J Appl Physiol.

[60] Dimitriadis K, Narkiewicz K, Leontsinis I, Konstantinidis D, Mihas C, Andrikou I, Thomopoulos C, Tousoulis D, Tsioufis K (2022). Acute effects of electronic and tobacco cigarette smoking on sympathetic nerve activity and blood pressure in humans. Int J Environ Res Public Health.

[61] Thijssen DH, Dawson EA, Tinken TM, Cable NT, Green DJ (2009). Retrograde flow and shear rate acutely impair endothelial function in humans. Hypertension.

[62] Hwang J, Ing MH, Salazar A, Lassegue B, Griendling K, Navab M, Sevanian A, Hsiai TK (2003). Pulsatile versus oscillatory shear stress regulates NADPH oxidase subunit expression: implication for native LDL oxidation. Circ Res.

[63] Ziegler T, Bouzourene K, Harrison VJ, Brunner HR, Hayoz D (1998). Influence of oscillatory and unidirectional flow environments on the expression of endothelin and nitric oxide synthase in cultured endothelial cells. Arterioscler Thromb Vasc Biol.

[64] Jiang T, Hu W, Zhang S, Ren C, Lin S, Zhou Z, Wu H, Yin J, Tan L (2022). Fibroblast growth factor 10 attenuates chronic obstructive pulmonary disease by protecting against glycocalyx impairment and endothelial apoptosis. Respir Res.

[65] Ikonomidis I, Marinou M, Vlastos D, Kourea K, Andreadou I, Liarakos N, Triantafyllidi H, Pavlidis G, Tsougos E, Parissis J, Lekakis J (2017). Effects of varenicline and nicotine replacement therapy on arterial elasticity, endothelial glycocalyx and oxidative stress during a 3-month smoking cessation program. Atherosclerosis.

[66] Ikonomidis I, Katogiannis K, Kourea K, Kostelli G, Pavlidis G, Thymus J, Katsanaki E, Maratou E, Lambadiari V (2023). Differential effects of heat-not-burn, electronic, and conventional cigarettes on endothelial glycocaly. Eur Heart J Imaging Methods Pract.

[67] Rao P, Han DD, Tan K, Mohammadi L, Derakhshandeh R, Navabzadeh M, Goyal N, Springer ML (2022). Comparable impairment of vascular endothelial function by a wide range of electronic nicotine delivery devices. Nicotine Tob Res.

[68] Jin L, Lynch J, Richardson A, Lorkiewicz P, Srivastava S, Theis W, Shirk G, Hand A, Bhatnagar A, Srivastava S, Conklin DJ (2021). Electronic cigarette solvents, pulmonary irritation, and endothelial dysfunction: role of acetaldehyde and formaldehyde. Am J Physiol Heart Circ Physiol.

[69] Mills A, Dakhlallah D, Robinson M, Kirk A, Llavina S, Boyd JW, Chantler PD, Olfert IM (2022). Short-term effects of electronic cigarettes on cerebrovascular function: a time course study. Exp Physiol.

[70] Pitzer CR, Aboaziza EA, O'Reilly JM, Mandler WK, Olfert IM (2023). Nicotine and microvascular responses in skeletal muscle from acute exposure to cigarettes and vaping. Int J Mol Sci.

[71] Ogunwale MA, Li M, Ramakrishnam Raju MV, Chen Y, Nantz MH, Conklin DJ, Fu XA (2017). Aldehyde detection in electronic cigarette aerosols. ACS Omega.

[72] Jensen RP, Luo W, Pankow JF, Strongin RM, Peyton DH (2015). Hidden formaldehyde in e-cigarette aerosols. N Engl J Med.

[73] Lee WH, Ong SG, Zhou Y, Tian L, Bae HR, Baker N, Whitlatch A, Mohammadi L, Guo H, Nadeau KC, Springer ML, Schick SF, Bhatnagar A, Wu JC (2019). Modeling cardiovascular risks of e-cigarettes with human-induced pluripotent stem cell-derived endothelial cells. J Am Coll Cardiol.

[74] Kerasioti E, Veskoukis AS, Skaperda Z, Zacharias A, Poulas K, Lazopoulos G, Kouretas D (2020). The flavoring and not the nicotine content is a decisive factor for the effects of refill liquids of electronic cigarette on the redox status of endothelial cells. Toxicol Rep.

[75] Fetterman JL, Weisbrod RM, Feng B, Bastin R, Tuttle ST, Holbrook M, Baker G, Robertson RM, Conklin DJ, Bhatnagar A, Hamburg NM (2018). Flavorings in tobacco products induce endothelial cell dysfunction. Arterioscler Thromb Vasc Biol.

[76] Ali FRM, Seidenberg AB, Crane E, Seaman E, Tynan MA, Marynak K (2023). E-cigarette unit sales by product and flavor type, and top-selling brands, United States, 2020–2022. MMWR Morb Mortal Wkly Rep.

[77] Havermans A, Krusemann EJZ, Pennings J, de Graaf K, Boesveldt S, Talhout R (2021). Nearly 20 000 e-liquids and 250 unique flavour descriptions: an overview of the Dutch market based on information from manufacturers. Tob Control.

